# Performance of a Receptive Language Test among Young Children in Madagascar

**DOI:** 10.1371/journal.pone.0121767

**Published:** 2015-04-01

**Authors:** Ann M. Weber, Lia C. H. Fernald, Emanuela Galasso, Lisy Ratsifandrihamanana

**Affiliations:** 1 Department of Psychology, Stanford University, Stanford, California, United States of America; 2 School of Public Health, University of California, Berkeley, California, United States of America; 3 Development Research Group, World Bank, Washington D.C., United States of America; 4 Le Centre Médico-Educatif “Des Orchidées Blanches”, Antananarivo, Madagascar; Middlesex University Dubai, UNITED ARAB EMIRATES

## Abstract

Language tests developed and validated in one country may lose their desired properties when translated for use in another, possibly resulting in misleading estimates of ability. Using Item Response Theory (IRT) methodology, we assess the performance of a test of receptive vocabulary, the U.S.-validated Peabody Picture Vocabulary Test-Third Edition (PPVT-III), when translated, adapted, and administered to children 3 to 10 years of age in Madagascar (N = 1372), in the local language (Malagasy). Though Malagasy is considered a single language, there are numerous dialects spoken in Madagascar. Our findings were that test scores were positively correlated with age and indicators of socio-economic status. However, over half (57/96) of items evidenced unexpected response variation and/or bias by local dialect spoken. We also encountered measurement error and reduced differentiation among person abilities when we used the publishers’ recommended stopping rules, largely because we lost the original item ordering by difficulty when we translated test items into Malagasy. Our results suggest that bias and testing inefficiency introduced from the translation of the PPVT can be significantly reduced with the use of methods based on IRT at both the pre-testing and analysis stages. We explore and discuss implications for cross-cultural comparisons of internationally recognized tests, such as the PPVT.

## Introduction

Interventions worldwide aim to improve developmental outcomes for young children [1, 2], generally targeting the negative correlates of poverty such as inadequate nutrition, health care, or education. Early child assessments used to examine the effectiveness of these interventions often focus on the language domain, since successful language skills are crucial to cognitive and socio-emotional development [3]. For example, early language skills predict later cognitive function among children in both high-income [4, 5] and low-income countries [6, 7]. Furthermore, language ability may be responsive to early intervention because it is a higher cognitive process that does not achieve adult levels of development until late in adolescence [8]. As children enter their preschool years, their knowledge and use of language can be measured directly using standardized assessment tools. We demonstrate in this paper that it is difficult to obtain an unbiased estimate of language ability in children from cultures that differ from where such tests were normed. The estimation process can be compromised by features of the measurement instrument, which can have important implications for the interpretation of results, and subsequently for the development of policy recommendations.

In the U.S. and other high-income countries (HIC), there are well-recognized and accepted guidelines that promote the sound and ethical use of standardized tests [9]. In a HIC context, stakes are high when test scores are used to determine placement in a private school or insurance coverage for a child’s developmental delay. As a result, great care is taken by publishers to develop “bias-free” tests, avoiding sources of bias that may result in systematically lower or higher scores for a given sub-group of respondents—such as a group defined by gender, ethnicity, or socio-economic status. However, most low- and middle-income countries (LMIC) do not have access to similarly “bias free” standardized tests, nor the professional organizations that set guidelines for avoiding bias in testing. And yet, the stakes are also high when tests are used to estimate the prevalence of developmental achievement or delay in a population, or to evaluate the success or failure of a program, which may influence the allocation of scarce resources or international aid.

The lack of well-validated instruments in LMIC makes the process of conducting language assessments problematic and even controversial. An ideal test would provide a valid index of a child’s “true” underlying language ability, regardless of where that child resides. Unfortunately, there is no simple way to ensure cross-cultural comparability of language tests. Since language skills are critically dependent on the linguistic and cultural context in which a child lives, there is an argument for every culture having its own specialized assessment method. Such an approach emphasizes the unique features of different cultures and the need to assess children in ways that respect their individual circumstances. However, by definition, this approach precludes direct comparisons of results across countries and ignores the need to assess whether children are given an equal chance of developing to their full potential. Consequently, this position neglects children’s universal rights to optimal development, as guaranteed in the Convention for the Rights of the Child [10].

Another position proposes that all children should be judged by exactly the same measurement, just as we assess global malnutrition and growth faltering with the WHO growth standards [11]. In keeping with this approach, UNICEF has examined child outcomes across multiple countries using the Multiple Indicator Cluster Survey (MICS) [12], with the aim of obtaining internationally comparable data to inform policy and program development, as well as to monitor progress toward national and international goals, including the Millennium Development Goals (MDGs). While useful in many ways, this approach fails to account for the wide range of different values and ways of learning that result in some abilities developing more quickly in some cultures than others. Importantly, local gold standard instruments typically do not exist to verify that such a “universal” test is actually measuring what is intended.

In the absence of locally validated measures, most investigators in LMIC take a position that falls somewhere in between the positions described above by adapting an existing test that has been shown to work in a similar research context, rather than designing a new test from scratch. As a consequence, well-established existing measures available in the U.S. or Europe are commonly adapted for use in other countries and translated to the local language [6, 13, 14]. The extent to which a test is modified to the local context will vary according to the needs of the study, to restrictions imposed by the test publisher, and to key constraints (expertise, time and money). The ideal choice of instrument varies with the study objectives and with the ease of adaptation and administration for the sample population of interest.

Here, we focus on a tool frequently selected for measuring receptive language skill: the Peabody Picture Vocabulary Test-Third Edition (PPVT-III) [15], a multi-item instrument that assesses receptive vocabulary in adults and children older than 2 ½ years. On each item, participants are asked to point to the correct image from a panel of four images in response to a stimulus word. The interviewer records whether or not the participant selects the correct image. The popularity of the PPVT is due in part to the wide age range that can be tested, the ease of test administration, the portability of the test, and the minimal amount of training required for testers. These features make the PPVT attractive in challenging field conditions and useful for longitudinal studies.

The PPVT was first created in 1959 for use in the U.S. and was based on extensive research on common English words, consultation with subject matter experts, and checks for reliability and validity; the first version of the test also established age and gender-appropriate standards using a representative sample of the U.S. population [16]. The PPVT is currently in its 4th revision; each revision attempting to reduce sources of bias by modifying items that favor certain U.S. subgroups over others. A Spanish version of the PPVT (the Test de Vocabulario en Imagines Peabody, or TVIP [17]) has been formally developed and widely used in Central and Latin America [18–21]. The PPVT has been administered throughout the world in many different languages with the purpose to assess effects of health and development interventions [7, 13, 22]. Although published norm-referenced scores for the PPVT do not apply outside of a U.S. sample, the PPVT has been used for cross-cultural research [19]. The repeated use of the PPVT across studies has been useful for comparing associations and trends; actual test scores cannot be compared, however.

In this paper, we review results from a large-scale nutrition study using form B of the PPVT (PPVT-IIIB) that was adapted and translated for use with Malagasy-speaking children living in rural villages throughout Madagascar. The PPVT data were obtained when children were 3–6 years and again when they were 7–10 years of age. We describe children’s performance on the PPVT at both time points, and the correlation of their scores with their basic demographics (age, gender, maternal education, and household wealth). However, since tests developed and carefully validated in one language and culture are not guaranteed to remain valid once the tests are adapted and translated for use in another setting [14, 23], our objective was to assess the extent and magnitude of bias in performance that may have been introduced. We used a combination of classical test and item response methods to explore overall test and item performance, as well as item-level bias by dialect spoken in the home. Specifically, we were concerned with the following three ways in which the internal validity of the test may have been compromised.

### Item difficulty & ordering

Our first concern was the loss of test item ordering by difficulty following the translation of the stimulus words, where English words would become harder or easier after translation to Malagasy. Within a series of 12 items in the PPVT, the easiest 3 items are designed to be given first and the hardest 3 last. The items in a given series increase in overall difficulty as the series number increases (e.g., all items in series 1 are designed to be easier than all items in series 2) [16]. Sequencing the items in this way permits the PPVT to be used across a wide range of ages when combined with rules that dictate age-appropriate starting and stopping rules. When start rules are used, respondents are credited for earlier items that were not administered, and when stop rules are used, subjects are not credited for later items. For both stopping and starting, the rules and test scores are based on retaining the order of item difficulty. We hypothesized that estimated ability in some children would be biased due to loss of item order difficulty in Malagasy when start or stopping rules were used.

### Item validity

Our second concern was the validity of the individual PPVT items and whether Malagasy children’s responses to those items matched our expectations for receptive vocabulary knowledge, the construct being measured. For example, we expect that children with larger vocabularies will have a higher probability of knowing hard words than children with small vocabularies. However, certain items may have been so unfamiliar in Madagascar that all children in our sample chose an image at random for these items. Another possibility was that certain items were known to children with small vocabularies but not to children with larger vocabularies due to differing usage of these words (or familiarity with the corresponding image) by some social, economic or geographic context. Therefore, we hypothesized that children would respond unpredictably to certain PPVT items in Malagasy and would fail to follow expected response patterns due to specific properties of these items, thus introducing error into our estimates.

### Subgroup biases

Our third concern was a threat to test validity from systematic differences that can occur when a test is administered to subgroups from different cultural or linguistic backgrounds within the same country or context. In some situations, subgroups may differ in their overall “true” mean ability, commonly referred to as differential impact [24], where observed group effects reflect a valid difference among subgroups. For example, children of highly-educated mothers may perform better on average on tests of intelligence than children of uneducated mothers [25]. A second type of subgroup difference can result from unfairness that occurs at the item level and is referred to as differential item functioning (DIF). DIF is a measure of whether test takers from different subgroups (e.g., gender) with the same underlying “true” ability give similar responses to an item on a test [24, 26]. For example, even though underlying ability is the same for boys and girls, more boys than girls may respond correctly to a test item using a sports analogy because boys may be more familiar with that particular sport. This would constitute DIF, or item bias, by gender. Summing over all items with DIF, the net effect may be that subgroups differ in their estimated mean ability if more items are easier for one group (e.g., boys score higher overall than girls on the hypothetical test). DIF may even exist if the direction of the bias varies by item and cancels out when items are added together (e.g., the same number of items favors girls as favors boys), which means that lack of a difference in mean ability by subgroup does not necessarily indicate an absence of DIF. As a result, DIF must be examined using specialized methods and cannot merely be controlled as a confounder with subgroup indicators in a regression analysis of a summary score.

Language spoken in the home is a factor that is frequently investigated for DIF with the PPVT in the U.S. [27], and was a serious concern in our study in Madagascar. Although Malagasy is considered a single language for translation purposes, there are numerous dialects spoken in Madagascar that are region-specific and vary by ethnic descent. The written Malagasy language is referred to as “official Malagasy” and is based on Merina, the dialect spoken in and around Antananarivo, the capital [28]. In six of the Malagasy dialects spoken by other ethnic groups, the lexical similarity with Merina is reportedly between 62 and 75% [29]. Since a single translation cannot account for the linguistic and cultural diversity in dialects, we hypothesized that certain items in the PPVT suffered from DIF by official Malagasy vs. non-Merina dialects spoken in Madagascar.

## Method

### Sample Data

We analyzed data from a study cohort established in 2004 when children were 0 to 3 years old, during an evaluation of a national nutrition program in Madagascar aimed at reducing the high prevalence of malnutrition in the country. First implemented in 1999 by the National Office of Nutrition (ONN), the ongoing program included over 5,550 sites with coverage of approximately 1.1 million children [30]. For the study, children living in 150 low-income communities from all six provinces in Madagascar were administered a battery of tests in 2007 (when children were 3 to 6 years) and again in 2011 (when they were 7 to 10 years). Many of the same interviewers were hired in 2007 and 2011, and received extensive classroom and field training prior to both surveys. Excellent inter-rater reliability (r > .95) was obtained during the training for the 2007 survey; inter-rater reliability for the 2011 survey was not available.

### Ethical Considerations

This project utilized anonymous and de-identified data from Madagascar and was approved by the Office for the Protection of Human Subjects at the University of California Berkeley.

### Malagasy Adaptation of the PPVT-IIIB

To develop the Malagasy version of the instrument, we translated all the stimulus words in the PPVT-III form B into official Malagasy and back-translated into English. In eight of the 96 items used in this study, no equivalent word exists in Malagasy and thus we used the French equivalent. These words are: kangaroo (item #15), horn (#20), ambulance (#33), panda (#38), dentist (#43), hyena (#75), walrus (#80), and tropical (#86). The French words for circle (item #10) and triangle (#39) were also used in 2007 because geometry is taught in French in Madagascar, and the Malagasy words were unfamiliar to children. However, these two words were changed to the Malagasy equivalent in 2011 when children were aged 7 to 10, so as not to favor children who were attending school. In both years, a few images were modified that were culturally inappropriate or ambiguous in the local context (e.g., a depiction of US dollars and cents was replaced with a picture of ariary and iraimbilanja, the Malagasy equivalents).

The published instruction manual recommends administering the PPVT in a sequential series of 12 items each, stopping if the respondent makes 8 or more mistakes in a series of 12. In 2007, all children were started at series 1 and continued through to series 6 or until they hit the stopping rule. Therefore, children in 2007 were administered a minimum of 12 items (from the first series) and a maximum of 72 items (all 6 series). In a preliminary analysis of the 2007 data, we found that translation had changed the ordering of the items by difficulty. We did not re-order the items because it is not allowed without permission by the publisher. In 2011, we selected six consecutive series to administer fully to all children without imposing start or stopping rules, since we knew that the items were not properly ordered. We selected item series numbers 3 to 8 as age-appropriate for a sample of Malagasy children aged 7 to 10 years; thus, 72 items were administered to all children in 2011. A subset of 48 overlapping items was administered in both years.

### Analytic Techniques

We examined the performance of the overall instrument and test items using Classical Test Theory (CTT) and Item Response Theory (IRT). CTT assumes that each person has a “true score” for a particular ability (or characteristic) that would have been obtained if the ability had been measured without error [31]. CTT methods are implemented primarily at the instrument level (e.g., the raw score total estimates ability) and ignore information available at the item level or shared information across respondents [24]. Item response models are probability models that take into account responses by all subjects in the sample to estimate any one person’s ability, as well as the performance of each item in the test. The theory underlying IRT-based models, also known as latent trait theory, is commonly used in education and psychology for assessing multi-item measures of latent constructs [32]. An important advantage of item response models (over the classical approach) is that they can be augmented to statistically investigate possible sources of bias at the item level, such as differential item functioning (DIF).

#### Classical Test Methods

We calculated raw score totals for all items administered to each subject, with one point given for every correctly identified word. Since all children were started from the same point (start rules were not used in either year), we did not credit children for non-administered items. For the raw score total, non-administered items that followed the stopping rule were scored as zero. We estimated evidence of internal consistency with the Cronbach’s alpha indicator [33]. We calculated pairwise Pearson or Spearman rank correlations between the total scores and several demographic characteristics, including mother’s education and household wealth [34]. We coded mother’s education as: no education (0), any primary level education (1), or secondary level education and above (2). We had previously generated the household wealth index using principal component analysis to aggregate wealth-related variables (i.e., asset ownership and dwelling characteristics such as electricity, running water, composition of floor, walls, and roof) into a single measure [35].

#### Item Response Methods

To assess the psychometric properties of the instrument, we applied the simplest statistical model in IRT to the data: the unidimensional Rasch model for dichotomous items (items scored as 0/1). In this model, the probability of an observed response is a function of the difference between person ability and item difficulty [32]. The Rasch principle of specific objectivity states that as long as the model holds, then we do not need any particular set of persons to obtain estimates of the item difficulties, nor do we need to give every person the same set of items to estimate their relative ability. This is particularly useful in situations with a lot of missing data due to non-response, or for groups of respondents administered a different set of items, both of which occurred in Madagascar. Therefore, we treated non-administered items as missing (not zero), such that the probability of correctly responding to a missing item was imputed by the Rasch model from non-missing responses by the child to other items of similar difficulty and non-missing responses to the item by other children of similar ability. By imposing the Rasch model, we accept that there is a monotonic relation between the person ability and item difficulty and the probability of a correct response in our data. We verified that this assumption was reasonably held by confirming that estimated ability on the PPVT was correlated with age.

We ran separate unidimensional Rasch models on the data at both time points and obtained item difficulty estimates for item numbers 1 to 72 in 2007 and items 25 to 96 in 2011, with two sets of item difficulties generated for the 48 overlapping items 25 to 72. For model identification, we constrained the mean of the item difficulty parameters to zero by setting the last item parameter to the negative sum of the other items. We constructed child ability estimates from expected *a-posteriori* (EAP) distribution, where the latent ability distribution was assumed to be Gaussian (M = 0, SD = 1). The EAP distribution refers to the expected value of the predicted distribution of scores for a given case, given the response pattern of that case and the estimated model parameters. Although EAP relies heavily on the distribution of the data and is sensitive to the sample population, it outperforms other estimation methods (such as maximum likelihood estimate) in situations where there is a substantial amount of missing data [36].

The mathematical unit of the Rasch model is the log-odds unit (logit) and is the same for person ability and item difficulty. We calculated standard errors (SE) of measurement for both the item difficulty and person ability parameter estimates. The SE for ability is a function of both ability and item properties, improving with increasing number of items administered to the respondent that are located near the respondent’s ability. Plots of ability estimates against SE have a U-shaped pattern if there is good overlap between the locations of the item difficulties and person abilities (i.e., errors increase for respondents with abilities at the extremes where there are fewer items that match their ability).

To test how well the responses to the items fit the statistical model, we used the weighted mean square (MNSQ) fit statistic, or infit [37], which is a ratio of the variances of the observed residuals over expected residuals for the model. An infit equal to one indicates that the observed residuals vary as much as would be expected by chance, infit values above one denote positive misfit, or more variation than expected, and infit values of less than one denote negative misfit, or less variation than expected. Some deviation from one is expected due to random error. We considered the infit to be acceptable if it fell between 0.75 and 1.33 (3/4 and 4/3) [37]. If a t-statistic (based on a transformation of the infit into a standard normal distribution) was greater than 2 or less than -2, we considered it to be evidence of statistically significant misfit [37]. We looked for patterns among items that demonstrated significant misfit and included item characteristic curves (ICC, i.e., plots of the probability of a correct response as a function of ability) for several items. Deviations of the empirical ICC (based on the observed data) from the modeled ICC are indicative of a lack of fit of the item.

Finally, we calculated person separation reliability as the difference in the observed total variance of the estimated abilities and the residual variance not explained by the model, divided by the variance explained by the model. Person separation reliability (or index) refers to the reproducibility of the location of the measure for one person relative to another. Low person separation reliability signifies that there is insufficient differentiation among person abilities to distinguish between them, either due to large measurement error or too narrow a range in abilities in the sample, given the test items administered. This measure of instrument reliability is expected to give comparable results to the Cronbach’s alpha obtained with classical methods. The Pearson correlation between the EAP estimates from the two years was disattenuated for measurement error by dividing the correlation by the square root of the product of the reliability coefficients of the two years [38, 39].

#### Differential Item Function

We assessed differential impact and differential item functioning for dialect spoken in the home. Due to the large number of dialects, we did not capture actual dialect spoken in the survey. We created a dichotomous indicator for language based on a question posed in 2011 to the child’s primary caregiver: “What language do you speak with your child at home: “official Malagasy”, “French”, or “a local dialect”? The indicator was set to zero if the primary caregiver reported that the language spoken was official Malagasy or to one if a local dialect was spoken. We imputed missing language information from the community median response. Since French was selected only twice, we replaced these observations with the community median.

We tested differential impact and DIF by adding two terms to the Rasch model: a term for dialect group membership (for impact) and an interaction term between each item and the group (for DIF). The parameter estimate obtained for group membership is an estimate of the overall mean difference in abilities between the subgroups. If this mean difference was more than 1.96 times its standard error, we considered the differential impact to be statistically significant. We estimated DIF effect size from the interaction term for each item and categorized effect sizes as negligible (< 0.426 logits), medium (≥ 0.426 and ≤ 0.638 logits), or large (> 0.638 logits) based on the system developed and used by the Educational Testing Service [40, 41]. Similarly to differential impact, we considered DIF of an item statistically significant if the DIF effect size was greater than 1.96 times the standard error of the DIF effect estimate. Items were considered to be exhibiting DIF if the DIF was found to be both statistically significant and the effect size was large. Some items had small to moderate DIF that was significant, or large DIF that was not significant, but we did not include these items in the DIF item counts discussed in this paper (unless otherwise noted).

### Sample Characteristics

Scores from the PPVT were available for a total of 1,372 children from 2007 or 2011. Data were available for 1,244 children in 2007, 1,224 children in 2011, and 1,096 children in both years. Just over half of the children assessed in either year were female (51.1%). The mean age was 54.6 months (SD = 10.4, range = 33–76) in 2007 and 103.2 months (SD = 10.5, range = 81–126) in 2011 (Table 1). Less than one fifth of the sample resided in urban areas (17.9%). Approximately 24% of the children’s mothers’ were uneducated, about half had some primary school education (55.6%), and fewer than 21% achieved secondary or above education.

**Table 1:** Summary demographics for the full sample.

Child characteristic (N = 1372)	% (unless noted)
Female	51.1
Mean age in '07 (SD, range)	54.6 (10.4, 33–76)
Mean age '11 (SD, range)	103.2 (10.5, 81–126)
Urban location	17.9
Maternal education, none	23.6
Maternal education, primary	55.6
Maternal education, ≥ secondary	20.8
Speaks local dialect other than official Malagasy	76.4
1^st^ Wealth Quintile	20.4
2^nd^ Wealth Quintile	19.7
3^rd^ Wealth Quintile	20.1
4^th^ Wealth Quintile	20.4
5^th^ Wealth Quintile	18.5
Antananarivo province	15.2
Fianarantsoa province	21.8
Toamasina province	17.6
Mahajanga province	11.9
Toliary province	27.4
Antsiranana province	6.1

Most children spoke a local dialect other than official Malagasy in their home (76.4%). Dialect spoken was almost entirely a function of province, with approximately 61% of official Malagasy speakers living in the capital province of Antananarivo and another 23% in the neighboring province of Fianarantsoa (data not shown). Dialect spoken was also a function of household socio-economic factors. Among the mothers with no education, the percentage of children who spoke a local dialect at home was nearly 92%. Among the poorest households (the bottom wealth quintile in the sample), the percentage of children who spoke a local dialect was 95% versus 58% in the wealthiest households (top wealth quintile).

### Software

We used ACER ConQuest version 2.0 for all of the IRT modeling [36] and Stata/MP 10.1 for Windows for obtaining the descriptive statistics.

## Results

### Respondent Performance

We present basic descriptive statistics for the raw total scores and Rasch model scores for 2007 and 2011 in Table 2. As expected, children’s raw scores increased with age (r = .31 in 2007 and .25 in 2011, both p < .001). Children successfully identified an average of approximately one additional word for every four months of increasing age in either period (i.e., the cross-sectional effect across different children of different ages in a given year). We could not directly estimate the average gain in ability over time from 2007 to 2011 (i.e., the longitudinal effect for the same children) as the scores were on different scales. However, the 2007 scores were positively correlated with those from 2011 (r = .42 for raw scores, p < .001). Correcting for attenuation by measurement error increased the correlation of the Rasch model estimates from .44 to .60 (p < .001).

**Table 2:** Comparison of Models.

	Raw Score (words)	Rasch (logits)
Items used to estimate ability	12–72 items	72 items
Mean ability in 2007 (median, SD, range)	13.6 (11, 8.7, 1 to 57)	-0.86 (-0.87, 0.49, -2.2 to 1.1)
Mean ability in 2011 (median, SD, range)	30.5 (29, 9.5, 11 to 60)	-0.37 (-0.46, 0.57, -1.7 to 1.4)
Correlation of ‘07 and ‘11 scores^†^	.42	.60
Median (range) 2007 Standard Error	N/A	0.37 (0.21–0.49)
Median (range) 2011 Standard Error	N/A	0.25 (0.24–0.30)
Separation Reliability 2007	N/A	.64
Separation Reliability 2011	N/A	.83

^†^ Raw and unidimensional Rasch score correlations are Pearson correlations.

The raw score correlation is not corrected for measurement error.

The two types of scores, raw total and Rasch model scores, were very highly correlated with each other (r ≥ .89 and p < .001 for both years, Table 3). The scores were also significantly correlated with mother’s education and household wealth index, with a larger positive correlation for the older cohort (Table 3). The Pearson correlation of the scores with maternal education increased from .21 in 2007 to .39 in 2011, and with household wealth from .35 in 2007 to .53 in 2011. Speaking a local dialect at home other than official Malagasy was negatively correlated with the scores (r = -.23 in 2007 and-.41 in 2011, both p < .001). Gender was not significantly correlated with the scores in either year (p ≥ .10).

**Table 3:** Correlations of test scores with demographics.

	2007 Scores		2011 Scores	
	Raw	Rasch	Raw	Rasch
**Rasch score**	.89	1	> .99	1
**Female**	-.02	-.02	-.05	-.05
**Speaks local dialect** ^**†**^	-.23	-.24	-.41	-.41
**Urban location**	.20	.20	.16	.16
**Maternal education** ^**†**^	.21	.21	.39	.39
**Household wealth (index)**	.35	.32	.53	.53

^†^ Local dialect is a binary indicator that the child spoke a dialect other than official Malagasy at home. Mother’s education was coded as ordinal categories: none (0), primary (1), or secondary and above (2).

**Fig 1:**
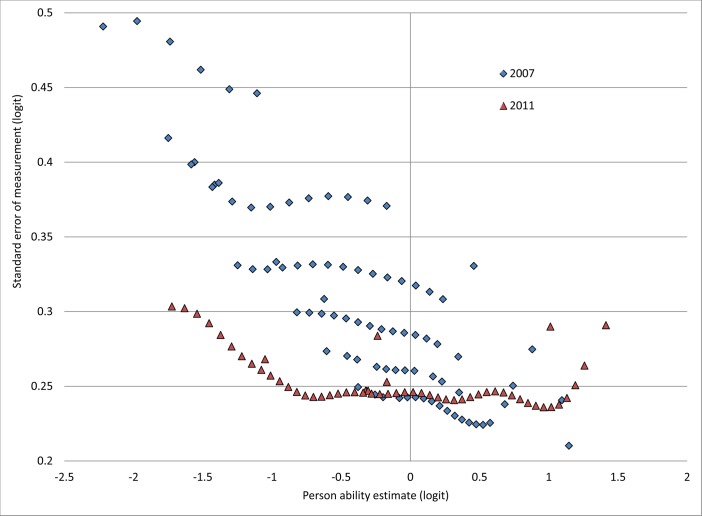
Standard error of measurement for the Rasch model ability estimates in 2007 and 2011. In 2007, the standard error on children’s estimated ability increased as the number of item sets administered to the children decreased. In 2011, the standard error on children’s estimated ability followed the expected pattern of increasing error at the extremes of low and high estimated ability.

We show the standard errors of measurement as a function of Rasch model ability estimates in Fig 1 for both years. In 2011, all children were administered 72 items and the errors followed the expected U-shaped pattern. However, in 2007, approximately 10% of the children completed only 12 items and another 54% were stopped after 24 items. Less than 5% of children completed series 6 (72 items). As a result, the errors increased as the amount of censoring increased in 2007. Children who had been administered all 72 items had the lowest standard errors of roughly 0.25 logits (based on Fig 1). This is equivalent to a 95% confidence interval (CI) of approximately one logit around their estimated ability (e.g., for an estimated ability of zero logit, the CI is -0.49 to 0.49 logits), or about a third of the full range of all abilities in 2007. For the approximately 10% of children who were stopped after the first series in 2007, the confidence intervals were nearly twice as large at 2 logits wide (CI of 2/3^rd^ of the range in abilities).

With nearly complete data for all 72 items administered in 2011, we estimated the internal consistency of responses to items at .85 using the Cronbach’s alpha coefficient. We do not report alpha for the set of items administered in 2007 as we had insufficient data for items that were not administered due to the use of stopping rules. In the Rasch model, the person separation reliability was moderate at .64 in 2007, and increased to .83 in 2011.

### Test Performance

#### Item difficulty & ordering

Item difficulty estimates were distributed from approximately -4 to 2 logits in 2007 and from -2.7 to 2.1 logits in 2011 (see S1 Table and S2 Table). To interpret the scale of the item difficulties (with mean zero), it is useful to think in terms of the relative location of person ability and item difficulty estimates, which are on the same scale. Based on the Rasch model, children with estimated ability above an item difficulty have a greater than 50% probability of answering that item correctly, and children with ability below an item difficulty have a less than 50% probability of answering correctly. In 2007, the mean child ability estimate was 0.86 logits below the mean item difficulty of zero, indicating that the items were too difficult on average. In 2011, the mean child ability estimate was -0.37 logits (close to zero) and the items were dispersed across the full range of person abilities, indicating that the item difficulties were age-appropriate (see S1 and S2 Figs).

In 2007, the standard error of measurement for the item difficulties ranged from 0.06 to 0.25 logits, with the exception of the last item (#72, SE = 1.2 logits), which was constrained to have a difficulty equal to the negative sum of the other items, and administered to < 5% of the sample. In 2011, the errors on the item estimates were much smaller on average, ranging from 0.05 to 0.06 logits, with the exception of the last item (#96, SE = 0.43 logits). The smaller standard errors are consistent with the larger number of responses in 2011 for all of the items.

As expected, the original ordering by item difficulty was lost in both years. For example, item #68 (tortoise) was easier than almost all of the other items preceding it, whereas item #29 (coin) was harder than most. In 2007, children who failed early items were therefore prevented from answering later, easy items due to the use of the stopping rule, and their raw total scores were censored.

#### Item validity

The weighted mean square fit statistics (infit) for all 96 items were within the acceptable boundaries of 0.75 and 1.33 (see S1 Table and S2 Table). However, the infit differed significantly from one for a number of items (t-statistic for infit < -2 or > 2), which we expected given the large number of items tested and the large sample size. Specifically in 2011, 18 out of 72 items exhibited statistically significant negative infit and another 13 items had statistically significant positive infit. We found that over a third of the items with significant negative infit were action verbs, with item #30 (peeking) having the largest negative infit in both years (t-statistic = -10). Seven of the eight French words that were administered to the children had significant positive infit in one of the two years of test administration.

**Fig 2:**
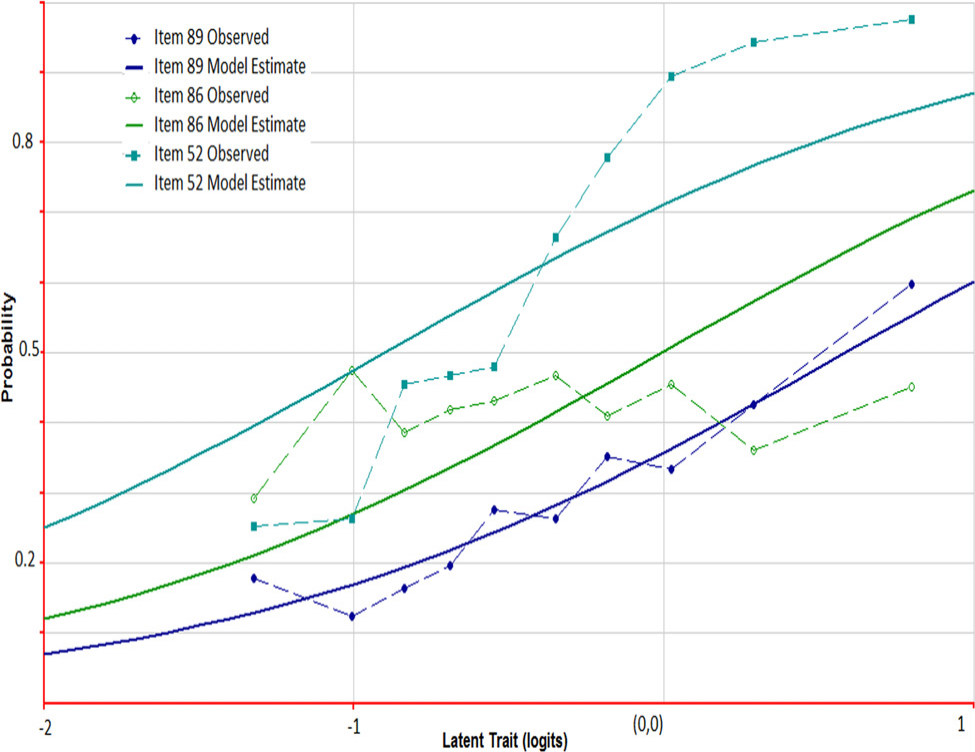
Item characteristic curves (ICC) in 2011 for items 52, 86 & 89. The curves represent the probability of a correct response to the indicated item as a function of ability of the respondent. Deviations of the empirical ICC (observed data) from the modeled ICC are indicative of a lack of fit of the item. For example, a flat curve for the observed data is evidence of positive infit (too much variation), and a curve with a steep transition from low to high ability is evidence of negative infit (too little variation). Item 89 (river) is an example of an item with excellent fit (observed data fits the model). Item 52 (huge) is an example with negative infit and item 86 (tropical) is an example with positive infit.

Examples of ICCs from 2011 are shown in Fig 2 for items #52, #86, and #89. Item #89 (river) was an example of an item with excellent fit (infit = 0.99, t-statistic = -0.3), where the probability of knowing the word increased as expected with estimated ability. Item #52 (huge) was an example of an item with significant negative infit (infit = 0.85, t-statistic = -7.8), and item #86 (tropical) was an example with significant positive infit (infit = 1.16, t-statistic = 8.2). The observed probabilities were flat across the range of person ability for item #86 (less discriminating), whereas item #52 had a steeper curve than expected by the model (more discriminating). In general, items exhibiting large and statistically significant negative infit are not a serious concern. However, items exhibiting positive infit contribute less toward the estimation of ability and are more problematic as they do not follow a predictable pattern of response [37].

#### Subgroup biases

In the 2007 model, the estimated differential impact by dialect was statistically significant at .27 logits (SE = 0.012), with children who spoke a local dialect other than official Malagasy scoring lower (on average) than those who spoke official Malagasy. In 2011, the differential impact by dialect was more than double that of 2007 at .68 logits (SE = 0.008), and statistically significant. Again, children who spoke a local dialect scored lower on average. These impact estimates were equivalent to about 8% and 22% of the full range of the ability estimates in 2007 and 2011, respectively. If we assume a range in raw scores of about 50 words in either year, this translates to an overall difference by dialect spoken of about 4 words in 2007 and 11 words in 2011.

At the item level, we found large and statistically significant DIF by dialect: 14 out of 72 items in 2007 and 27 out of 72 items in 2011, respectively. The absolute value of the magnitude of the DIF effect was as high as 1.82 logits. Nine items in each year had a DIF effect size that exceeded plus or minus one logit (~30% of the full range of scores). Nearly half the items with dialect DIF favored those who spoke official Malagasy and just over half favored a local dialect. Added together, the net DIF effect from these items favored children who spoke a local dialect by 0.88 logits in 2007 and 0.19 logits in 2011 (standard errors not calculated).

**Fig 3:**
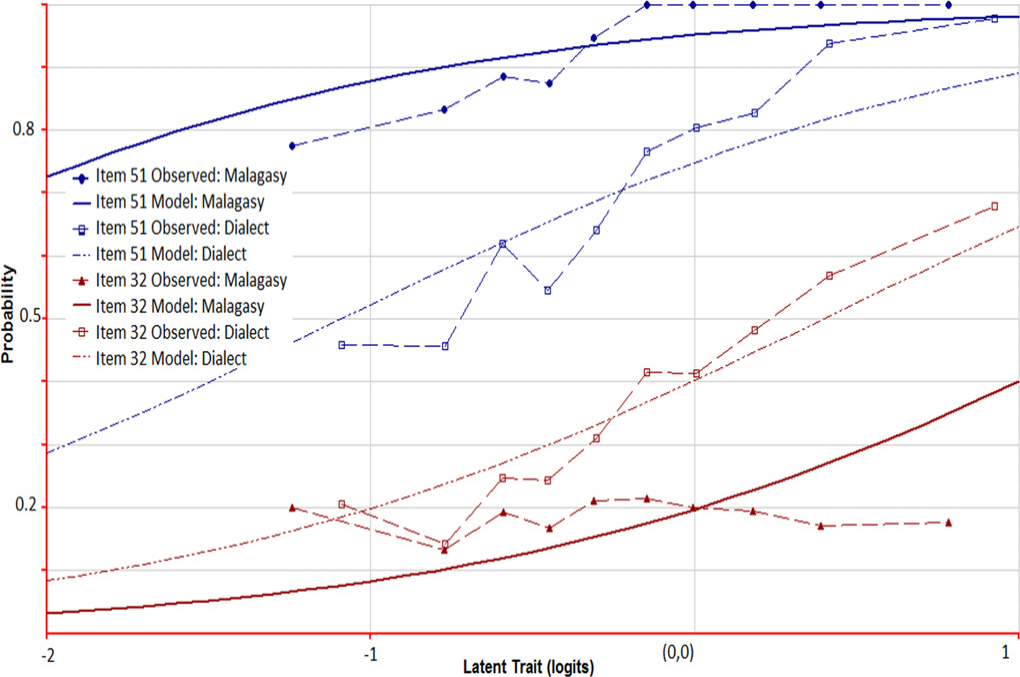
Item characteristic curves for items 32 & 51 with dialect DIF in 2011. The ICC for item 51 (jogging) is indicative of an item that favored children who spoke official Malagasy. The probability of success is higher for this group (curve labeled “Item 51 Model: Malagasy”) than for the group who spoke a local dialect (curve labeled “Item 51 Model: Dialect”). Item 32 (goat) is an example of an item that favored a local dialect. Item 32 also demonstrated significant positive infit for the children who spoke official Malagasy as shown by the deviation of the empirical ICC (curve labeled “Item 32 Observed: Malagasy”) from the modeled ICC (curve labeled “Item 32 Model: Malagasy”).

Among the items in common for both years, we found substantial variation in which of the items exhibited DIF, with only 4 items exhibiting DIF by dialect in both years. Item characteristic curves in 2011 for items #32 and #51 by dialect spoken are shown in Fig 3. Item #51 (jogging) is an example that favored children who spoke official Malagasy (the probability of success is higher for this group as shown with the solid blue curve in Fig 3, labeled “Item 51 Model: Malagasy”). Item #32 (goat) is an example of an item that favored a local dialect and also exhibited positive infit. There are two words for goat in Madagascar: one used in the central highlands where the official dialect is spoken, and the other used in the remainder of the country. The word used outside of the region of the official dialect was used in the translation and, as a consequence, children who spoke official Malagasy found this word harder (estimated DIF effect is 1.1 logits in 2001 and 1.7 logits in 2011). The observed responses from children who knew the word for item #32 visually followed the modeled probability curve (curves labeled “Item 32 Model: Dialect” and “Item 32 Observed: Dialect”). On the other hand, the observed responses from the children who didn’t know the word resulted in a flat curve indicating positive infit (curve labeled “Item 32 Observed: Malagasy”).

The items that favored children who spoke a local dialect included 7 of the 8 French words with no Malagasy equivalent (e.g., panda). The group of items favoring official Malagasy contained some relatively easy vocabulary words that are used commonly in everyday life (e.g. cat, baby, broom, and bottle). Items administered towards the end of the test, as well as the Malagsy words for geometric shapes, were less likely to evidence dialect DIF, possibly because children were equally tired at the end of the test or equally unfamiliar with the words. Although we can speculate on possible explanations for other specific words (e.g., the item for “dressing” shows a child putting on socks, but in the coastal areas children walk barefoot or in sandals), no additional patterns were found that might explain dialect DIF.

We investigated differences in the PPVT by gender, but the mean difference was very small in both years with only 6 out of 72 items exhibiting significant DIF by gender in 2007 and none in 2011. Given the lack of DIF by gender, these data are not discussed further here.

## Discussion

Malagasy children’s overall performance on the PPVT was consistent with results reported by others who have translated and adapted the PPVT to non-English speaking, low-income countries [19, 42]. Specifically, children’s total scores trended upwards with age and were positively and significantly correlated with socio-economic factors represented by maternal education and household wealth. However, as hypothesized, we found that the psychometric properties of the PPVT were not maintained in Madagascar, threatening the validity of the test in this context.

First, we lost the original ordering of items by difficulty established by the publisher. As others have noted, item difficulty equivalence is not guaranteed by achieving linguistic equivalence given that there can be important dialectical, cultural and geographic differences in frequency of word use [14, 23, 43]. In Madagascar, the loss of order of item difficulties combined with the use of stopping rules in 2007 prevented children from demonstrating their knowledge of easy words that occurred in later series of the PPVT, biasing their scores downward. In addition, we found that items administered in 2007 were too hard on average, such that low-scoring children were not administered enough easy items to obtain a precise estimate of their ability, and our capacity to distinguish among these children was diminished. In 2011, we improved the precision of our estimates and the reliability of the instrument by starting at a series where the item difficulties were age-appropriate and by administering 72 items to all children. Other authors have chosen to re-order items after translation in order to retain stopping rules and avoid bias from changes to item difficulty ordering [44].

Second, nearly half of the items behaved poorly in terms of expected fit to our model, either adding noise to ability estimates because there was no association between ability and the difficulty of the item (i.e., positive infit); or contributing information—but only over a very narrow range of abilities because the transition from not knowing the item to knowing the item was very steep (i.e., negative infit). Although these noisy and non-informative items can be dropped in the analysis phase [19], administering twice as many items (as we did in 2011) was extremely inefficient and likely led to test fatigue.

Finally, in both years, the test scores suffered from non-random error introduced by 37 items that were systematically easier or harder depending on the dialect spoken in the home. Simple everyday words tended to be easier for children who spoke official Malagasy. This may be explained by the fact that Malagasy dialects share roughly 70% of their lexicons with official Malagasy [29]. We suspect that everyday words may be part of the percentage that do not overlap, in that synonyms for these words are used more commonly in local dialects than the official words administered in our test. On the other hand, children who spoke a local dialect performed better with the French words. Prior to this analysis, we thought that knowledge of French would be associated with wealth and living in the central highlands near the capital (where official Malagasy is spoken). However, the observed direction of bias from using French words was the reverse. We now speculate that coastal dialects may have incorporated French or foreign-sounding words into their vocabulary when Madagascar was colonized. In our sample, nearly all the children on the coast were categorized as speaking a local dialect. As with other sources of measurement error, bias from DIF can be avoided by replacing or dropping problematic items [19]. In the recommendation section, we discuss these and additional options to handling DIF.

After we identified the items with differential functioning by dialect and quantified the DIF size for each, we added these error estimates together and found that the net effect of item bias favored children who spoke a local dialect. This is opposite to what we found for the overall mean impact of dialect on the total scores: children who spoke a local dialect at home performed worse on average than those who spoke official Malagasy. Children who spoke a local dialect were also from poorer households and were more likely to have less educated mothers. The moderate positive correlation of the scores with mother’s education and household wealth provides a probable explanation for this observed mean impact. We can rule out that the impact of dialect is entirely due to DIF, since the net bias favored children who spoke a local dialect, but these same children performed worse overall on the test. However, the inclusion of the items with DIF by dialect in the scoring likely resulted in an underestimate of the true difference in vocabulary knowledge between the 2 dialectical groups. Assuming dialect spoken in the home is an indicator of socio-economic status (SES) in Madagascar, then DIF by dialect will also bias our estimates of SES impact towards no difference. In a separate analysis, we found that the difference in vocabulary score between children in the lowest SES households versus the highest SES households was moderate to large (Cohen’s d = 0.7) [45]. Unfortunately, we cannot obtain a precise estimate of the overall magnitude of the bias due to measurement error in estimating DIF at the item level.

## Recommendations

Based on our findings, we offer several recommendations when an existing instrument, such as the PPVT, has been translated and adapted for use in a study in a low- or middle-income country. In the preparation phase, we suggest pre-testing many more items for an instrument than are thought to be necessary, without the use of start or stopping rules, to identify item ordering and items with poor fit or DIF. Ideally, the publisher would allow the removal, replacement, or modification of problem items. For example, in our study, the French words (i.e., stimulus words without a Malagasy equivalent) introduced random error into our estimates of ability and/or were biased in favor of children who spoke a local dialect. The replacement of these French words with words from one of the distracter images that had a Malagasy equivalent would have eliminated this problem [44]. Testing many more items than necessary has the additional benefit of determining how well the item difficulties cover the full range of person abilities (necessary for obtaining low standard errors of ability) and identifying how many items are needed to obtain high person separation reliability, to differentiate well among children. For example, we had poor overlap of the item difficulties with children’s abilities in 2007. In retrospect, we could have improved reliability and precision in 2007 by adding (and testing) new items with difficulties that matched children’s abilities in the bottom half of the distribution and dropping very hard items.

Once problem items have been addressed, the new set of items should be re-tested in an iterative process until no additional problems are identified. The final items can then be ordered from easiest to hardest, and stopping rules implemented to avoid test fatigue. Although recommended by test publishers, we do not support using rules that allow for starting mid-test and working backwards to find a basal set (the set of items below which the respondent will make no errors). This technique is difficult to train and implement properly in large-scale studies because of the high probability of human error during the administration of the test.

If modifications to the instrument are not allowed by the publisher (i.e., re-ordering or changing stimulus words), then we recommend identifying a minimum set of consecutive items with reasonable person separation reliability and administering this set of items to the full sample without the use of start or stopping rules, as we did in 2011. However, we could have improved the validity and efficiency of the test and reduced test fatigue if we had skipped or changed items that in the best case provided little extra information for estimating the children’s abilities (redundant or highly discriminating), and in the worst case biased our results.

Although not ideal, there are options in the analysis phase for handling items that introduce bias from DIF. First, items with DIF can be ignored. If the net bias is zero when adding up the DIF effect of multiple items, then the consequences of choosing to do nothing may not be serious. However, if the total DIF effect favors one group over another, then the overall ability estimates will be biased, as is the case in our sample. A second option is to perform separate analyses by subgroup. Researchers from the Young Lives study used this option when items were flagged with DIF by language [19]. This approach might be costly in terms of loss of power in the analysis. More importantly, it would change the research question, as the analysis could not be applied to the entire sample. A third option is to drop the problem items from the analysis. The investigators of the Young Lives study also reported and excluded items with gender DIF from their analysis [19, 46]. Although this option seems reasonable, there are tradeoffs. Some DIF may be due to chance alone and items would be dropped unnecessarily and result in loss of reliability. In addition, one form of validity (at the item level) may be improved while another form of validity (at the instrument level) may be lost. The underlying construct being measured may not be the same (this also applies if items are dropped or replaced in pre-testing). The fourth option is to incorporate an interaction term for the item and group membership into the item response model when estimating person ability. This may be the best option, although the interpretation of person ability becomes complicated as the number of subgroups increase.

In future work, we will use a combination of the first and third options for handling items with poor performance. Items that are highly discriminating, with less variation than expected, will be left in the estimation process—they do no harm and may improve reliability. A cut-point or specific criteria will be chosen for dropping items with the strongest evidence of random error and the largest DIF effect size for dialect spoken at home. The criteria will be set so as to maximize the use of items and the reliability, while minimizing sources of item-level bias.

For all of the above, we recommend the use of IRT methods over the more common reliance on classical methods. CCT methods lack the detailed statistics for item difficulty available from IRT methods that are useful for assessing whether to modify, drop, or re-order items. In addition, the classical approach does not provide standard errors on the ability estimates, which can inform whether individuals should be dropped from the analysis and whether a sufficient number of items were administered for a given ability level. Despite the advantages associated with IRT methods, their use is uncommon in many fields. We have found limited evidence of the use of IRT in other studies where measures, such as the PPVT, are used outside the cultural and linguistic contexts of their original design.

## Conclusion

Just in the last decade, the WHO has reshaped how we think about children’s growth potential around the world. The 2006 standards for height and weight set the bar for “how all children should grow rather than merely describing how children grew at a specified time and place” [47]. Instead of being limited to comparisons within a population, we now compare growth across populations and advocate for changes to policies or programs. Obtaining a similar set of international standards for language could likewise be a powerful tool for change. Research in early language development is occurring across cultures and countries [19], and thus the need already exists for tools that allow for making valid international comparisons [48].

Modifying and internally validating an existing measure to the local context while maintaining its desired psychometric properties may offer a way forward. We suggest a hybrid approach to the extremes of a single universal instrument vs. a set of unique locally-developed instruments. Although the majority of items could be unique to the local context for best reliability and internal validity, the hybrid would include a subset of universal items that anchor the test scores for international comparisons. Importantly, the success of optimizing an instrument for a given context will depend on being able to change, drop, add, and re-arrange items as necessary. Validated information gathered from different countries and languages could then be made available to other interested parties [19, 46]. For example, USAID’s Education for Decision Making (EdData II) has developed two instruments, EGMA and EGRA, that have been applied in 44 countries and in 80 languages [49]. Interested parties can download complete instruments and manuals in several languages, along with guidance for adaptations, and reports from studies conducted around the world. Similarly, an extensive library of items could be made available for receptive vocabulary. In this model, investigators would choose culturally and age-appropriate items for their study. A group of experts would be needed to manage the library, the data, the protocols for structuring and validating the test, as well as to provide support for new users—not a small task.

This paper makes clear the scientific risk to inference that can occur when otherwise careful and responsible researchers neglect to carefully validate a translation of an instrument that was originally developed in a different language. This failure can occur due to the absence of a local gold standard for external validation, the researchers’ lack of familiarity with the methods used to establish internal validity, or restrictions on instrument modification placed on the user by the publisher. Unfortunately, the absence of carefully validated measures in low- and middle-income countries can result in misleading estimates of children’s language ability. This has significant policy and funding implications for programs or services in those countries and hinders valid cross-country comparisons. More importantly, decisions made based on these estimates have consequences for the children the programs are trying to help.

## Supporting Information

S1 Fig Wright Map for the unidimensional IRT model of all children, all items in 2007. The histogram on the left hand side of the figure illustrates the distribution of person ability in 2007 (each 'X' represents 3.9 cases). The item difficulties in 2007 are located on the right hand side at the point where a respondent has a 50% chance of responding correctly to the item. Persons with abilities above the threshold have a greater than 50% chance of getting the item right and persons below the threshold have less than a 50% chance. A logit difference between item difficulty and person ability of +1 is equivalent to a probability of .73 of responding correctly to the item, and a logit difference of -1 is equivalent to a probability of .27. A look at the map shows: a) that the original ordering by item difficulty was lost (e.g., item #68 (tortoise) was easier than almost all of the other items preceding it, whereas item #29 (coin) was harder than most), b) that the distribution of person abilities is approximately normally distributed, and c) that the items were too difficult on average as indicated by the fact that the mean child ability estimate was 0.86 logits below the mean item difficulty of zero.
(DOCX)

S2 Fig Wright Map for the unidimensional IRT model of all children, all items in 2011. The histogram on the left hand side of the figure illustrates the distribution of person ability in 2011 (each 'X' represents 2.6 cases). The item difficulties in 2011 are located on the right hand side at the point where a respondent has a 50% chance of responding correctly to the item. Persons with abilities above the threshold have a greater than 50% chance of getting the item right and persons below the threshold have less than a 50% chance. A look at the map shows: a) that the original ordering by item difficulty was lost (as it was in 2007), b) that the distribution of person abilities is slightly skewed, although approximately normally distributed, and c) that the item difficulties were age-appropriate as indicated by the fact that the mean child ability estimate was close to zero and the items were dispersed across the full range of person abilities.
(DOCX)

S1 Table Item statistics from 2007 for the unidimensional model. Statistics for each item from 2007 include: the item number, difficulty estimate, standard error of measurement (SEM), weighted mean square (MNSQ) fit statistic (infit), t-statistic based on a transformation of the infit into a standard normal distribution, and a yes/no indicator of whether the item evidenced statistically significant differential item function by dialect spoken in the home. A t-statistic greater than 2 or less than -2 is evidence of statistically significant misfit.
(DOCX)

S2 Table Item statistics from 2011 for the unidimensional model. Statistics for each item from 2011 include: the item number, difficulty estimate, standard error of measurement (SEM), weighted mean square (MNSQ) fit statistic (infit), t-statistic based on a transformation of the infit into a standard normal distribution, and a yes/no indicator of whether the item evidenced statistically significant differential item function by dialect spoken in the home. A t-statistic greater than 2 or less than -2 is evidence of statistically significant misfit.
(DOCX)
