# Supplementary material for: Performance of a Receptive Language Test among Young Children in Madagascar
Source: PLoS One. 2015 Apr 1;10(4):e0121767. doi: 10.1371/journal.pone.0121767 (PMC4382173; doi:10.1371/journal.pone.0121767)
Supplement: S1 Fig — The histogram on the left hand side of the figure illustrates the distribution of person ability in 2007 (each 'X' represents 3.9 cases). The item difficulties in 2007 are located on the right hand side at the point where a respondent has a 50% chance of responding correctly to the item. Persons with abilities above the threshold have a greater than 50% chance of getting the item right and persons below the threshold have less than a 50% chance. A logit difference between item difficulty and person ability of +1 is equivalent to a probability of .73 of responding correctly to the item, and a logit difference of -1 is equivalent to a probability of .27. A look at the map shows: a) that the original ordering by item difficulty was lost (e.g., item #68 (tortoise) was easier than almost all of the other items preceding it, whereas item #29 (coin) was harder than most), b) that the distribution of person abilities is approximately normally distributed, and c) that the items were too difficult on average as indicated by the fact that the mean child ability estimate was 0.86 logits below the mean item difficulty of zero. (DOCX) [file pone.0121767.s001.docx]

**S1 Figure. Wright Map for the unidimensional IRT model of all children, all items in 2007.**

The histogram on the left hand side of the figure illustrates the distribution of person ability in 2007 (each 'X' represents 3.9 cases). The item difficulties in 2007 are located on the right hand side at the point where a respondent has a 50% chance of responding correctly to the item. Persons with abilities above the threshold have a greater than 50% chance of getting the item right and persons below the threshold have less than a 50% chance. A logit difference between item difficulty and person ability of +1 is equivalent to a probability of .73 of responding correctly to the item, and a logit difference of -1 is equivalent to a probability of .27. A look at the map shows: a) that the original ordering by item difficulty was lost (e.g., item #68 (tortoise) was easier than almost all of the other items preceding it, whereas item #29 (coin) was harder than most), b) that the distribution of person abilities is approximately normally distributed, and c) that the items were too difficult on average as indicated by the fact that the mean child ability estimate was 0.86 logits below the mean item difficulty of zero.

+item

---------------------------------------------------------------------------------------

|29 38 63 67 72 |

|54 |

| |

|57 |

| |

X|18 35 46 53 |

| |

1 |40 64 66 |

X|31 45 49 |

| |

X|13 32 33 |

|20 25 28 62 |

X|12 39 50 |

XX|58 70 |

X|43 56 |

X|44 65 |

XX|42 |

XXX|24 52 |

XXXXX|14 16 48 59 |

0 XXX|15 |

XXXXX|9 19 |

XXXXXXXX|23 26 41 61 |

XXXXXXXX|22 30 60 |

XXX|3 55 69 |

XXXXXXXXXXXXXXXXX|11 71 |

XXXXXXXX| |

XXXXXXXXXXXXXXXXXXXXX|5 36 |

XX|47 |

XXXXXXXXXXXXXXXXXXXXXXXXXXXXXXXXX| |

XXXXXXXXX|37 |

XXXXXXXXXXXXXXXXXXXXXXXXXXXXXXXXXXXXXXXX| |

-1 XXXXXXXXXXXXXXXXXXXXXXXXXXXXXXXXXXX| |

XX| |

XXXXXXXXXXXXXXXXXXXXXXXXXXXXXXXXXXXXX|17 |

X| |

XXXXXXXXXXXXXXXXXXXXXXXX|6 21 34 |

XXXXXXXX| |

| |

XXXXXXXXXXXXXXXXXXXXXXX| |

|27 51 |

XXXXXXXXX| |

| |

|68 |

XXX|10 |

-2 |7 |

|4 |

|8 |

| |

| |

| |

| |

| |

| |

|1 |

| |

| |

-3 | |

| |

| |

|2 |

=======================================================================================
