# Supplementary material for: Performance of a Receptive Language Test among Young Children in Madagascar
Source: PLoS One. 2015 Apr 1;10(4):e0121767. doi: 10.1371/journal.pone.0121767 (PMC4382173; doi:10.1371/journal.pone.0121767)
Supplement: S2 Fig — The histogram on the left hand side of the figure illustrates the distribution of person ability in 2011 (each 'X' represents 2.6 cases). The item difficulties in 2011 are located on the right hand side at the point where a respondent has a 50% chance of responding correctly to the item. Persons with abilities above the threshold have a greater than 50% chance of getting the item right and persons below the threshold have less than a 50% chance. A look at the map shows: a) that the original ordering by item difficulty was lost (as it was in 2007), b) that the distribution of person abilities is slightly skewed, although approximately normally distributed, and c) that the item difficulties were age-appropriate as indicated by the fact that the mean child ability estimate was close to zero and the items were dispersed across the full range of person abilities. (DOCX) [file pone.0121767.s002.docx]

**S2 Figure. Wright Map for the unidimensional IRT model of all children, all items in 2011.**

The histogram on the left hand side of the figure illustrates the distribution of person ability in 2011 (each 'X' represents 2.6 cases). The item difficulties in 2011 are located on the right hand side at the point where a respondent has a 50% chance of responding correctly to the item. Persons with abilities above the threshold have a greater than 50% chance of getting the item right and persons below the threshold have less than a 50% chance. A look at the map shows: a) that the original ordering by item difficulty was lost (as it was in 2007), b) that the distribution of person abilities is slightly skewed, although approximately normally distributed, and c) that the item difficulties were age-appropriate as indicated by the fact that the mean child ability estimate was close to zero and the items were dispersed across the full range of person abilities.

+item

---------------------------------------------------------------------------------------

|83 |

2 | |

| |

| |

| |

|80 |

| |

|29 72 |

X|94 |

|53 82 96 |

X|76 |

X|38 |

XX|58 |

1 XX|63 90 |

XXXXX|46 57 75 |

XXX|35 45 70 |

XXXXXX|54 73 |

XXXXXX| |

XXXXXXXXXX|77 89 |

XXXXXXX|43 62 |

XXXXXXX|31 32 |

XXXXXXXXXXXXXXXXX|33 74 79 |

XXXXXXXXXX|67 85 87 |

XXXXXXXXXXXXXXXXXXXX|49 66 |

XXXXXXXXX|26 |

0 XXXXXXXXX|44 86 |

XXXXXXXXXXXXXXXXXXXXXXXXXXXXXXXX|92 |

XXXXXXXXXXXXXX|30 50 78 |

XXXXXXXXXXXXXXXXXXXXXXXXXXXXXXXXXX|40 95 |

XXXXXXXXXXXXXXXX|65 81 |

XXXXXXXXXXXXXXXXX|61 64 91 |

XXXXXXXXXXXXXXXXXXXXXXXXXXXXXXXXXXX|39 56 |

XXXXXXXXXXXXXXXXXXXXXXXX|93 |

XXXXXXXXXXXXXXXXXXXXXXXXXXXXXXXXXXXXXXXX|42 59 60 |

XXXXXXXXXXXXXXXXXXXX|28 |

XXXXXXXXXXXXXXXXXXXX|88 |

XXXXXXXXXXXXXXXXXXXXXXXXXXXXXXXXXXXXX|52 69 |

-1 XXXXXXXXXXXXXXXX|25 84 |

XXXXXXXXXXXXXXXXXX|36 41 55 |

XXXXXXXXXXXXXXX| |

XXXXXXXXXXXX| |

XXXX|47 48 |

XX| |

XX| |

|37 51 |

| |

X| |

|71 |

| |

-2 | |

|68 |

|34 |

| |

| |

| |

| |

| |

|27 |

| |

| |

=======================================================================================
