# Supplementary material for: Performance of a Receptive Language Test among Young Children in Madagascar
Source: PLoS One. 2015 Apr 1;10(4):e0121767. doi: 10.1371/journal.pone.0121767 (PMC4382173; doi:10.1371/journal.pone.0121767)
Supplement: S1 Table — Statistics for each item from 2007 include: the item number, difficulty estimate, standard error of measurement (SEM), weighted mean square (MNSQ) fit statistic (infit), t-statistic based on a transformation of the infit into a standard normal distribution, and a yes/no indicator of whether the item evidenced statistically significant differential item function by dialect spoken in the home. A t-statistic greater than 2 or less than -2 is evidence of statistically significant misfit. (DOCX) [file pone.0121767.s003.docx]

**S1 Table: Item statistics from 2007 for the unidimensional model.**

Statistics for each item from 2007 include: the item number, difficulty estimate, standard error of measurement (SEM), weighted mean square (MNSQ) fit statistic (infit), t-statistic based on a transformation of the infit into a standard normal distribution, and a yes/no indicator of whether the item evidenced statistically significant differential item function by dialect spoken in the home. A t-statistic greater than 2 or less than -2 is evidence of statistically significant misfit.

|  | **2007: Unidimensional Rasch Model** | | | |  |
| --- | --- | --- | --- | --- | --- |
| **item #** | **Estimate** | **SEM** | **infit** | **t-statistic** | **Lang DIF** |
| 1 | -2.765 | 0.08 | 0.97 | -0.5 | yes |
| 2 | -4.14 | 0.129 | 0.97 | -0.2 | no |
| 3 | -0.335 | 0.06 | 0.96 | -2 | no |
| 4 | -2.132 | 0.067 | 0.96 | -1.1 | no |
| 5 | -0.606 | 0.058 | 1.02 | 1.4 | no |
| 6 | -1.28 | 0.059 | 0.97 | -1.5 | no |
| 7 | -2.057 | 0.066 | 0.94 | -1.8 | yes |
| 8 | -2.214 | 0.069 | 0.97 | -0.9 | no |
| 9 | -0.056 | 0.062 | 1.1 | 4.1 | no |
| 10 | -1.924 | 0.064 | 1.03 | 0.9 | no |
| 11 | -0.397 | 0.059 | 0.96 | -2.2 | yes |
| 12 | 0.543 | 0.07 | 0.96 | -0.9 | no |
| 13 | 0.717 | 0.075 | 0.96 | -1 | no |
| 14 | 0.095 | 0.066 | 0.96 | -1.5 | no |
| 15 | -0.037 | 0.064 | 1.13 | 5.2 | no |
| 16 | 0.108 | 0.066 | 0.96 | -1.6 | no |
| 17 | -1.136 | 0.062 | 1.03 | 1.8 | no |
| 18 | 1.104 | 0.084 | 1.06 | 1 | no |
| 19 | -0.101 | 0.064 | 1.05 | 2.1 | no |
| 20 | 0.671 | 0.074 | 1.01 | 0.4 | no |
| 21 | -1.302 | 0.062 | 0.97 | -1.3 | no |
| 22 | -0.295 | 0.062 | 1 | 0.1 | no |
| 23 | -0.172 | 0.063 | 1.01 | 0.3 | no |
| 24 | 0.162 | 0.066 | 0.99 | -0.2 | no |
| 25 | 0.651 | 0.11 | 0.98 | -0.3 | no |
| 26 | -0.165 | 0.1 | 0.98 | -1 | no |
| 27 | -1.617 | 0.114 | 1.02 | 0.3 | no |
| 28 | 0.624 | 0.109 | 0.95 | -1.1 | no |
| 29 | 1.998 | 0.155 | 1.03 | 0.2 | no |
| 30 | -0.22 | 0.1 | 0.92 | -3.5 | no |
| 31 | 0.89 | 0.115 | 1.02 | 0.3 | no |
| 32 | 0.714 | 0.111 | 1.02 | 0.4 | yes |
| 33 | 0.686 | 0.111 | 1.02 | 0.4 | no |
| 34 | -1.284 | 0.107 | 0.97 | -0.5 | no |
| 35 | 1.083 | 0.12 | 1.01 | 0.2 | no |
| 36 | -0.546 | 0.1 | 1.01 | 0.3 | no |
| 37 | -0.788 | 0.144 | 1 | 0.1 | yes |
| 38 | 2.044 | 0.192 | 1.08 | 0.5 | yes |
| 39 | 0.578 | 0.144 | 1.01 | 0.2 | no |
| 40 | 0.968 | 0.152 | 1.03 | 0.4 | no |
| 41 | -0.153 | 0.138 | 0.98 | -0.7 | no |
| 42 | 0.252 | 0.14 | 0.97 | -0.9 | no |
| 43 | 0.399 | 0.142 | 1.01 | 0.3 | yes |
| 44 | 0.325 | 0.141 | 1.03 | 0.7 | no |
| 45 | 0.845 | 0.15 | 0.99 | -0.1 | no |
| 46 | 1.12 | 0.157 | 1.03 | 0.3 | no |
| 47 | -0.698 | 0.143 | 0.99 | -0.2 | no |
| 48 | 0.052 | 0.139 | 0.99 | -0.4 | no |
| 49 | 0.882 | 0.187 | 1.04 | 0.5 | no |
| 50 | 0.579 | 0.181 | 1.03 | 0.4 | no |
| 51 | -1.601 | 0.213 | 0.93 | -0.3 | no |
| 52 | 0.156 | 0.178 | 0.99 | -0.3 | no |
| 53 | 1.103 | 0.192 | 1.03 | 0.3 | no |
| 54 | 1.478 | 0.203 | 1.07 | 0.5 | no |
| 55 | -0.309 | 0.18 | 0.98 | -0.4 | yes |
| 56 | 0.389 | 0.179 | 0.97 | -0.7 | no |
| 57 | 1.283 | 0.197 | 1.12 | 0.9 | no |
| 58 | 0.436 | 0.18 | 1 | -0.1 | no |
| 59 | 0.065 | 0.178 | 0.97 | -0.8 | yes |
| 60 | -0.261 | 0.18 | 0.95 | -1 | no |
| 61 | -0.162 | 0.206 | 0.98 | -0.3 | yes |
| 62 | 0.626 | 0.207 | 1.04 | 0.6 | no |
| 63 | 1.885 | 0.237 | 1.13 | 0.6 | yes |
| 64 | 0.93 | 0.211 | 1.05 | 0.5 | yes |
| 65 | 0.339 | 0.205 | 0.97 | -0.5 | no |
| 66 | 0.93 | 0.211 | 1.02 | 0.2 | yes |
| 67 | 1.549 | 0.226 | 1.13 | 0.7 | no |
| 68 | -1.839 | 0.251 | 0.93 | -0.2 | yes |
| 69 | -0.311 | 0.208 | 0.95 | -0.7 | no |
| 70 | 0.481 | 0.205 | 1.02 | 0.4 | no |
| 71 | -0.387 | 0.209 | 0.98 | -0.2 | no |
| 72 | 1.546 | 1.216 | 1.12 | 0.7 | no |
