# Supplementary material for: Performance of a Receptive Language Test among Young Children in Madagascar
Source: PLoS One. 2015 Apr 1;10(4):e0121767. doi: 10.1371/journal.pone.0121767 (PMC4382173; doi:10.1371/journal.pone.0121767)
Supplement: S2 Table — Statistics for each item from 2011 include: the item number, difficulty estimate, standard error of measurement (SEM), weighted mean square (MNSQ) fit statistic (infit), t-statistic based on a transformation of the infit into a standard normal distribution, and a yes/no indicator of whether the item evidenced statistically significant differential item function by dialect spoken in the home. A t-statistic greater than 2 or less than -2 is evidence of statistically significant misfit. (DOCX) [file pone.0121767.s004.docx]

**S2 Table*:* Item statistics from 2011 for the unidimensional model.**

Statistics for each item from 2011 include: the item number, difficulty estimate, standard error of measurement (SEM), weighted mean square (MNSQ) fit statistic (infit), t-statistic based on a transformation of the infit into a standard normal distribution, and a yes/no indicator of whether the item evidenced statistically significant differential item function by dialect spoken in the home. A t-statistic greater than 2 or less than -2 is evidence of statistically significant misfit.

|  | **2011: Unidimensional Rasch Model** | | | |  |
| --- | --- | --- | --- | --- | --- |
| **item #** | **Estimate** | **SEM** | **infit** | **t-statistic** | **Lang DIF** |
| 25 | -1.005 | 0.049 | 0.88 | -5.8 | no |
| 26 | 0.054 | 0.048 | 0.94 | -2.9 | no |
| 27 | -2.687 | 0.061 | 0.98 | -0.2 | yes |
| 28 | -0.744 | 0.048 | 0.93 | -3.9 | yes |
| 29 | 1.529 | 0.058 | 1.02 | 0.4 | no |
| 30 | -0.19 | 0.048 | 0.84 | -10.3 | yes |
| 31 | 0.41 | 0.05 | 0.95 | -1.9 | no |
| 32 | 0.44 | 0.05 | 1.08 | 2.9 | yes |
| 33 | 0.354 | 0.049 | 1.11 | 4.5 | yes |
| 34 | -2.178 | 0.057 | 0.95 | -0.9 | yes |
| 35 | 0.871 | 0.052 | 0.97 | -0.9 | no |
| 36 | -1.088 | 0.049 | 0.92 | -3.5 | yes |
| 37 | -1.579 | 0.052 | 0.97 | -0.9 | no |
| 38 | 1.18 | 0.055 | 1.08 | 1.8 | yes |
| 39 | -0.468 | 0.048 | 0.98 | -1.2 | no |
| 40 | -0.236 | 0.048 | 0.99 | -0.6 | no |
| 41 | -1.092 | 0.049 | 0.96 | -1.6 | no |
| 42 | -0.705 | 0.048 | 0.92 | -4.6 | no |
| 43 | 0.526 | 0.05 | 0.96 | -1.5 | no |
| 44 | 0.018 | 0.048 | 0.98 | -1.2 | no |
| 45 | 0.809 | 0.052 | 0.96 | -1.3 | yes |
| 46 | 0.937 | 0.053 | 1.1 | 2.5 | no |
| 47 | -1.323 | 0.05 | 0.91 | -3.3 | yes |
| 48 | -1.348 | 0.05 | 0.94 | -2 | no |
| 49 | 0.131 | 0.049 | 0.97 | -1.3 | yes |
| 50 | -0.167 | 0.048 | 0.96 | -2.2 | no |
| 51 | -1.612 | 0.052 | 0.91 | -2.6 | yes |
| 52 | -0.9 | 0.049 | 0.85 | -7.8 | yes |
| 53 | 1.31 | 0.056 | 1.08 | 1.7 | yes |
| 54 | 0.751 | 0.052 | 1.05 | 1.5 | no |
| 55 | -1.096 | 0.049 | 0.97 | -1.5 | no |
| 56 | -0.493 | 0.048 | 1.01 | 0.8 | no |
| 57 | 0.945 | 0.053 | 1.05 | 1.3 | yes |
| 58 | 1.117 | 0.054 | 1.06 | 1.5 | no |
| 59 | -0.669 | 0.048 | 0.96 | -2.6 | no |
| 60 | -0.687 | 0.048 | 0.95 | -2.9 | yes |
| 61 | -0.389 | 0.048 | 1.06 | 3.8 | yes |
| 62 | 0.473 | 0.05 | 0.99 | -0.3 | no |
| 63 | 0.995 | 0.053 | 0.95 | -1.2 | no |
| 64 | -0.406 | 0.048 | 1 | -0.3 | no |
| 65 | -0.318 | 0.048 | 0.98 | -1.5 | no |
| 66 | 0.141 | 0.049 | 0.94 | -2.9 | no |
| 67 | 0.281 | 0.049 | 1.14 | 6 | yes |
| 68 | -2.073 | 0.056 | 0.94 | -1.3 | yes |
| 69 | -0.916 | 0.049 | 0.86 | -7.5 | no |
| 70 | 0.859 | 0.052 | 1.01 | 0.3 | no |
| 71 | -1.81 | 0.053 | 0.93 | -1.9 | yes |
| 72 | 1.478 | 0.057 | 1.1 | 1.8 | yes |
| 73 | 0.732 | 0.051 | 1.02 | 0.7 | no |
| 74 | 0.326 | 0.049 | 1.13 | 5.2 | no |
| 75 | 0.927 | 0.053 | 1.1 | 2.6 | yes |
| 76 | 1.229 | 0.055 | 1.01 | 0.2 | no |
| 77 | 0.59 | 0.051 | 1.02 | 0.8 | no |
| 78 | -0.142 | 0.048 | 1.05 | 3 | no |
| 79 | 0.302 | 0.049 | 1.05 | 2.3 | no |
| 80 | 1.682 | 0.059 | 1.05 | 0.8 | no |
| 81 | -0.327 | 0.048 | 0.98 | -1.1 | no |
| 82 | 1.37 | 0.056 | 1.07 | 1.3 | no |
| 83 | 2.079 | 0.063 | 1.08 | 1 | yes |
| 84 | -0.986 | 0.049 | 0.98 | -0.8 | no |
| 85 | 0.22 | 0.049 | 1.01 | 0.6 | no |
| 86 | -0.006 | 0.048 | 1.16 | 8.2 | yes |
| 87 | 0.233 | 0.049 | 1.13 | 5.8 | yes |
| 88 | -0.832 | 0.048 | 0.9 | -5.7 | no |
| 89 | 0.587 | 0.051 | 0.99 | -0.3 | no |
| 90 | 1.012 | 0.053 | 1.11 | 2.6 | yes |
| 91 | -0.377 | 0.048 | 0.95 | -2.8 | no |
| 92 | -0.053 | 0.048 | 0.94 | -3.2 | no |
| 93 | -0.549 | 0.048 | 1.05 | 2.8 | yes |
| 94 | 1.424 | 0.057 | 1.01 | 0.1 | no |
| 95 | -0.259 | 0.048 | 0.93 | -4.3 | no |
| 96 | 1.386 | 0.43 | 1.07 | 1.4 | no |
